# Gut Metabolites Are More Predictive of Disease and Cohoused States than Gut Bacterial Features in a Polycystic Ovary Syndrome-Like Mouse Model

**DOI:** 10.1128/mSystems.01149-20

**Published:** 2021-09-14

**Authors:** Bryan Ho, Daniel Ryback, Basilin Benson, Cayla N. Mason, Pedro J. Torres, Robert A. Quinn, Varykina G. Thackray, Scott T. Kelley

**Affiliations:** a Bioinformatics and Medical Informatics Program, San Diego State University, San Diego, California, USA; b Department of Biology, San Diego State University, San Diego, California, USA; c Collaborative Mass Spectrometry Innovation Center, University of California San Diego, La Jolla, California, USA; d Skaggs School of Pharmacy and Pharmaceutical Sciences, University of California, San Diego, La Jolla, California, USA; e Department of Obstetrics, Gynecology and Reproductive Sciences, University of California, San Diego, La Jolla, California, USA; Colorado State University

**Keywords:** bile acids, bioinformatics, gut microbiome, longitudinal, metabolomics, metagenomics, mouse model, multiomics

## Abstract

Polycystic ovary syndrome (PCOS) impacts ∼10% of reproductive-aged women worldwide. In addition to infertility, women with PCOS suffer from metabolic dysregulation which increases their risk of developing type 2 diabetes, cardiovascular disease, and nonalcoholic fatty liver disease. Studies have shown differences in the gut microbiome of women with PCOS compared to controls, a pattern replicated in PCOS-like mouse models. Recently, using a letrozole (LET)-induced mouse model of PCOS, we demonstrated that cohousing was protective against development of metabolic and reproductive phenotypes and showed via 16S amplicon sequencing that this protection correlated with time-dependent shifts in gut bacteria. Here, we applied untargeted metabolomics and shotgun metagenomics approaches to further analyze the longitudinal samples from the cohousing experiment. Analysis of beta diversity found that untargeted metabolites had the strongest correlation to both disease and cohoused states and that shifts in metabolite diversity were detected prior to shifts in bacterial diversity. In addition, log_2_ fold analyses found numerous metabolite features, particularly bile acids (BAs), to be highly differentiated between placebo and LET, as well as LET cohoused with placebo versus LET. Our results indicate that changes in gut metabolites, particularly BAs, are associated with a PCOS-like phenotype as well as with the protective effect of cohousing. Our results also suggest that transfer of metabolites via coprophagy occurs rapidly and may precipitate changes in bacterial diversity. This study joins a growing body of research linking changes in primary and secondary BAs to host metabolism and gut microbes relevant to the pathology of PCOS.

**IMPORTANCE** Using a combination of untargeted metabolomics and metagenomics, we performed a comparative longitudinal analysis of the feces collected in a cohousing study with a PCOS-like mouse model. Our results showed that gut metabolite composition experienced earlier and more pronounced differentiation in both the disease model and cohoused mice compared with the microbial composition. Notably, statistical and machine learning approaches identified shifts in the relative abundance of primary and secondary BAs, which have been implicated as modifiers of gut microbial growth and diversity. Network correlation analysis showed strong associations between particular BAs and bacterial species, particularly members of *Lactobacillus*, and that these correlations were time and treatment dependent. Our results provide novel insights into host-microbe relationships related to hyperandrogenism in females and indicate that focused research into small-molecule control of gut microbial diversity and host physiology may provide new therapeutic options for the treatment of PCOS.

## INTRODUCTION

Polycystic ovary syndrome (PCOS), a common reproductive endocrine disorder, is estimated to affect ∼5 to 15% of reproductive-age women worldwide (1). PCOS is the most prevalent cause of anovulatory infertility, and women with this disorder have a higher risk of pregnancy-related complications (2). The diagnosis of PCOS is based on the 2003 Rotterdam criteria, which require two out of three of the following: hyperandrogenism, oligomenorrhea or amenorrhea, and polycystic ovaries (3). Although the precise etiology of PCOS is unknown, genetic and twin studies indicate that PCOS is a polygenic heritable disorder that is influenced by environmental factors including exposure to excess maternal androgens during fetal development (4–6). The onset of PCOS often occurs during the early reproductive years, indicating that puberty may be a critical period in the development of PCOS (7).

In addition to its effects on reproductive health, PCOS increases the risk of developing metabolic diseases such as type 2 diabetes, hypertension, and nonalcoholic fatty liver disease (NAFLD) (8, 9). Metabolic dysregulation manifests predominantly in women with PCOS who have hyperandrogenism and is independent of body mass index (10, 11). Alongside metabolic dysregulation, PCOS is also associated with changes in the gut microbiome (12, 13). The gut microbiome comprises a complex community of microorganisms that are important for host physiology including immunity, metabolism, and neurology (14). Gut microbes play a critical role in the fermentation of dietary fibers, synthesis of vitamins such as B_12_, modification of bile acids (BAs), neurotransmitters, and hormones, and production of short-chain fatty acids that regulate energy homeostasis (14). Dysbiosis of the gut microbiome has been correlated with multiple metabolic disorders, including obesity, type 2 diabetes, and NAFLD (15). With regard to PCOS, studies have shown that gut bacterial species richness is lower and that the relative abundance of specific bacterial taxa is altered in women with PCOS compared to women without the disorder (12, 13, 16–23). Furthermore, studies have demonstrated a strong correlation between gut microbial diversity or the abundance of specific gut bacterial taxa and hyperandrogenism, indicating that testosterone may modulate the composition of the gut microbiome in women (12, 13, 16–19, 22, 23).

Investigations of the gut microbiome in a letrozole (LET)-induced PCOS-like mouse model have also indicated a strong relationship between hyperandrogenism and shifts in the alpha diversity and composition of the gut microbiome (24, 25). This mouse model utilizes letrozole, a nonsteroidal aromatase inhibitor, to limit the conversion of testosterone to estrogen, resulting in increased testosterone and decreased estrogen levels. This model recapitulates many reproductive and metabolic hallmarks of PCOS including oligo- or anovulation, polycystic ovaries, elevated luteinizing hormone (LH) levels, weight gain, abdominal adiposity, dysglycemia, hyperinsulinemia, and insulin resistance (24–26). The importance of hyperandrogenism in this activational model was further demonstrated in a study that discontinued letrozole treatment in mice and demonstrated a recovery in reproductive, metabolic, and gut microbial phenotypes (27). A letrozole-induced PCOS-like rat model was also reported to have changes in the gut microbiome (28, 29).

Although correlative evidence from both human and rodent model studies indicates that there is an association between PCOS and the gut microbiome, a direct role of the gut microbiome in generating or exacerbating metabolic dysregulation in this disease state has yet to be established. Indeed, despite the many studies that have identified correlations between metabolic disorders such as obesity, type 2 diabetes, and NAFLD and shifts in the gut microbiome, very few have demonstrated a direct effect of gut microbes in these disorders (30). One method for establishing a causal link between the gut microbiome and host is via fecal microbiota transplant (FMT) into germfree mice. For example, Ridaura et al. transplanted stool samples from lean and obese human donors into germfree mice and found that the mice developed the donors’ metabolic phenotype (31). More recently, Qi et al. performed an FMT of stool from women with PCOS versus controls into antibiotic-depleted mice and showed that the FMT with PCOS stool was sufficient to result in a PCOS-like phenotype that included increased LH, acyclicity, polycystic ovaries, and insulin resistance (20). Since rodents are coprophagic, another approach is to perform a cohousing study. This method of horizontal transmission has been shown to result in an exchange of microbiota between caged individuals (32). Cohousing with healthy mice was reported to be protective against developing obesity and maternal high-fat-diet-induced metabolic dysregulation (31, 33). Altogether, these studies suggest that the gut microbiome may play a causative role in the development of metabolic disorders including PCOS.

**FIG 1:**
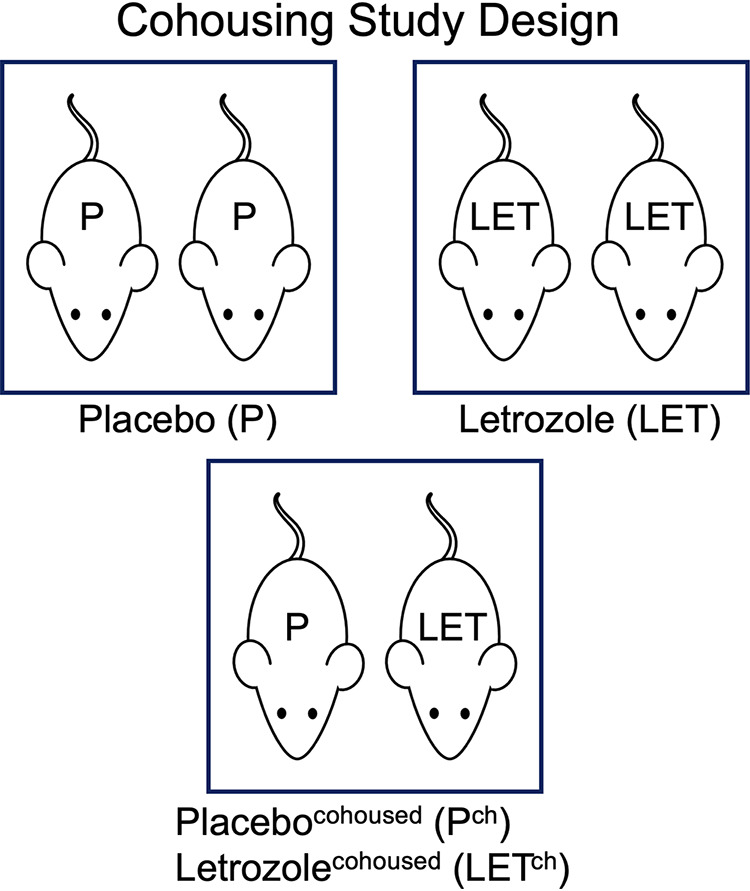
For the cohousing study design, mice implanted with either placebo or 3-mg letrozole pellets were housed two per cage in three separate housing arrangements. This resulted in four treatment groups: placebo mice (P), letrozole mice (LET), placebo cohoused with letrozole (P^ch^), and letrozole cohoused with placebo (LET^ch^) (*n* = 8 mice/group).

Recently, we performed a cohousing study using the letrozole mouse model to test whether exposure to a healthy gut microbiome was protective against developing PCOS metabolic or reproductive phenotypes (26). During the cohousing study, mice implanted with either placebo or letrozole pellets were housed two per cage in three separate housing arrangements (Fig. 1). This resulted in four treatment groups: placebo mice (P), letrozole mice (LET), placebo cohoused with letrozole (P^ch^), and letrozole cohoused with placebo (LET^ch^). This study demonstrated that cohousing with P mice improved both reproductive and metabolic phenotypes associated with letrozole treatment (26). Interestingly, gut microbial 16S rRNA sequencing analysis showed that the gut microbiome of LET^ch^ mice did not resemble the gut microbiome of P mice but instead was more similar to that of P^ch^ mice (26). These results suggested that cohousing resulted in an exchange of gut microbes and that transfer of the gut microbiome from a P mouse to a LET mouse was sufficient to provide protection from developing both metabolic and reproductive phenotypes of PCOS despite the lack of similarity between P and LET^ch^ mice.

In the present study, we applied multiple ‘omics approaches to further explore the longitudinal relationship of the gut microbiome samples collected in the letrozole-induced PCOS-like mouse model cohousing study (22). Although little is known about the relationship between gut metabolites and PCOS, a recent study by Qi et al. showed that the secondary BAs glycodeoxycholic acid and tauroursodeoxycholic acid (TUDCA) were present in lower abundance in a cohort of women with PCOS than in women without this disorder and that supplementation with these BAs was protective against developing a PCOS-like phenotype in mice (20). Given these results, we hypothesized that gut metabolites might provide greater explanatory power for longitudinal patterns in the data than gut microbes and that the combination of multiple ‘omics data sets might strengthen the time-dependent patterns previously observed with the 16S data. Specifically, we applied untargeted mass spectrometry (MS) analysis to identify the diversity of small molecules, including BAs, in the fecal samples and shotgun metagenomic sequencing to improve species- and strain-level identification of bacterial species.

## RESULTS

### Metabolites clustered by treatment in a time-dependent manner.

**FIG 2:**
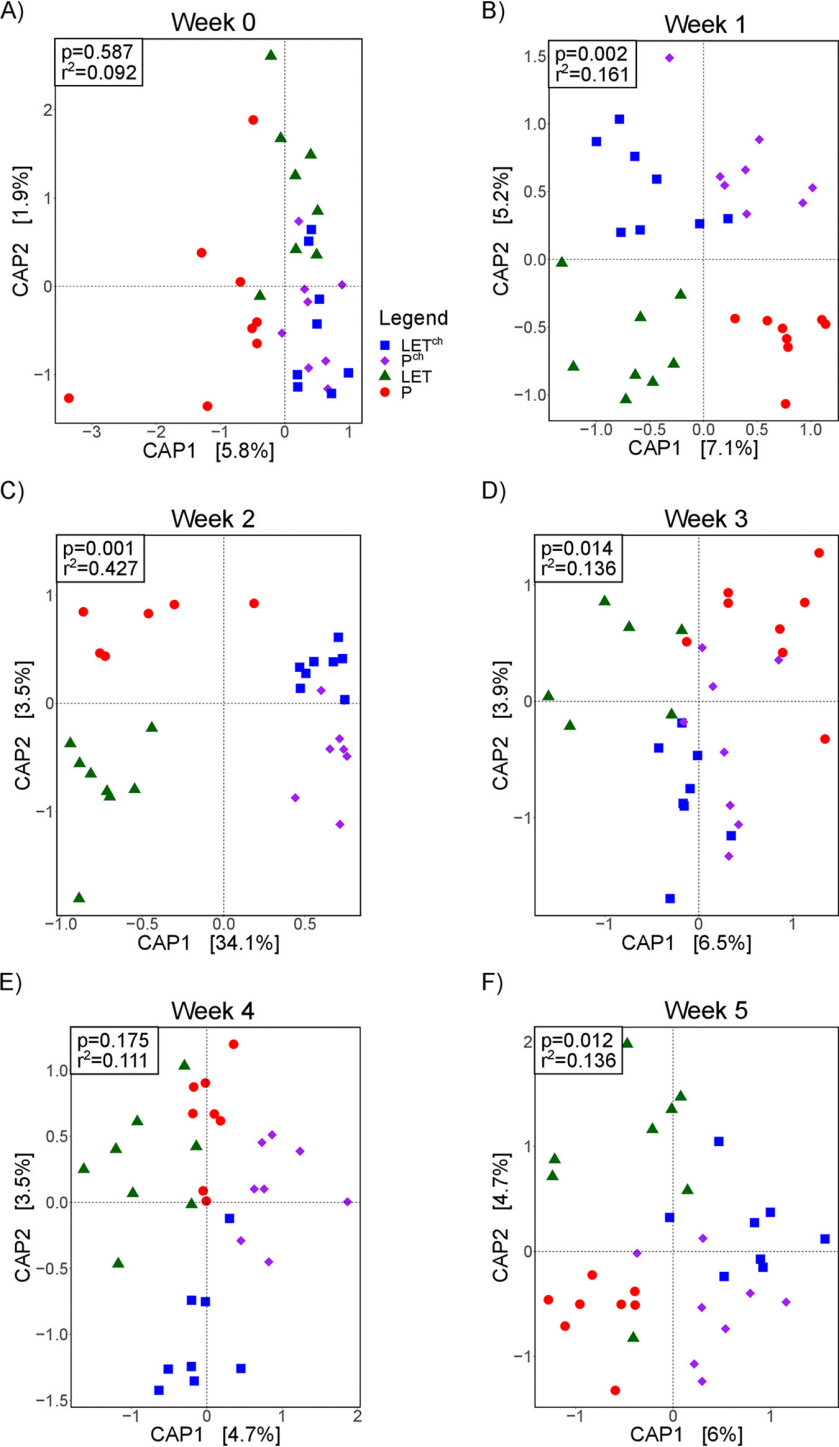
Cohousing letrozole-treated mice with placebo-treated mice influenced the overall composition of gut microbial metabolites over time. The cohousing study resulted in four treatment groups: placebo mice (P), letrozole mice (LET), placebo cohoused with letrozole (P^ch^), and letrozole cohoused with placebo (LET^ch^). (A to F) Constrained canonical analysis of principal (CAP) coordinates (Bray-Curtis distances) of fecal metabolites from the four treatment groups for weeks 0 to 5 posttreatment. Shown are the CAP1 and CAP2 coordinates, representing the two coordinates that captured the greatest amount of variation (percentage of variation is shown in brackets). Results of permutational ANOVA (PERMANOVA) of the Bray-Curtis distances are shown for each time point.

A CAP (canonical analysis of principal coordinates) analysis of untargeted metabolomic data generated from weekly fecal samples from the cohousing study found the degree of clustering associated with treatment differed substantially over the course of the study (Fig. 2). No treatment-associated clustering was observed based on sample metabolite composition prior to pellet implantation (week 0) (Fig. 2A). By week 1, we observed differentiation among samples from different treatments (Fig. 2B; *P* = 0.002, *R*^2^ = 0.161), with virtually no overlap among samples from the four treatment groups. The greatest degree of clustering occurred among the week 2 samples (Fig. 2C; *P* = 0.001, *R*^2^ = 0.427). At week 2, P and LET samples showed distinct separation from each other and from the cohousing samples (P^ch^ and LET^ch^) which clustered together. The amount of variation explained by the first two principal components was also highest at week 2 (Fig. 2C). We continued to observe significant associations between treatment and metabolites at week 3, but this was not observed at week 4 (Fig. 2D).

### Comparisons of beta diversity analyses across multiomics data sets found strongest association between metabolites and treatment.

**FIG 3:**
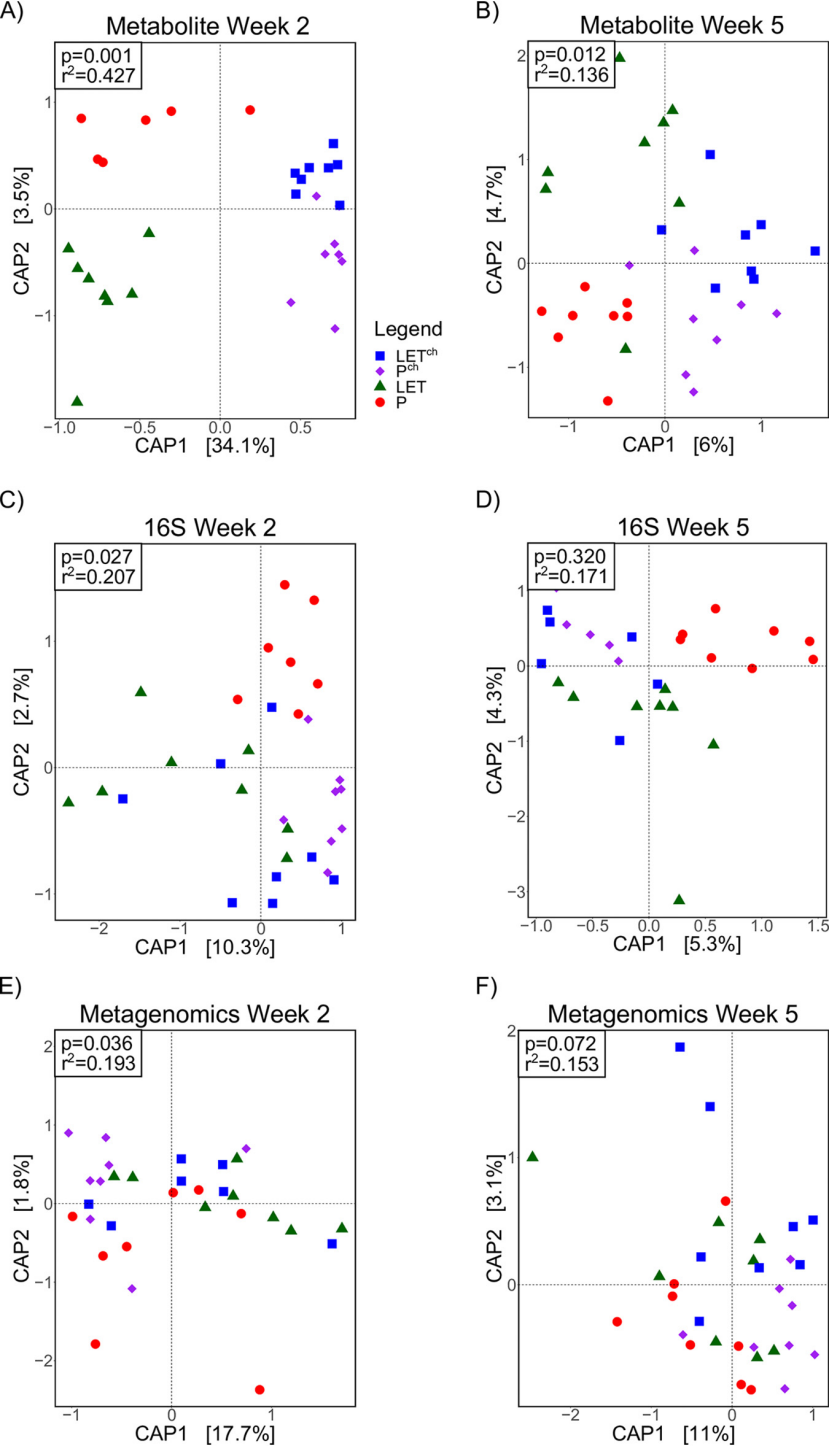
Cohousing letrozole with placebo mice resulted in greater differentiation among treatment groups in the overall composition of both gut microbes and metabolites at 2 weeks compared with 5 weeks. The cohousing study resulted in four treatment groups: placebo mice (P), letrozole mice (LET), placebo cohoused with letrozole (P^ch^), and letrozole cohoused with placebo (LET^ch^). Canonical analysis of principal (CAP) coordinates of Bray-Curtis dissimilarity among the four treatment groups, week 2 (A, C, E) and week 5 (B, D, F). Shown are the CAP1 and CAP2 coordinates, representing the two coordinates that captured the greatest amount of variation (percentage of variation is shown in brackets). (A and B) Metabolites; (C and D) bacterial 16S rRNA gene sequences (16S); (E and F) bacterial whole-genome sequencing (WGS).

The metabolite CAP analysis was compared with metagenomic and 16S rRNA CAP analyses of the gut microbiome at weeks 2 and 5, the time points for which we had all three data sets. At week 2, all three data sets showed clear differentiation among the samples from the four treatment groups (Fig. 3). The goodness of fit (*R*^2^) between data and treatment was highest for the metabolites (Fig. 3A; *P* = 0.001, *R*^2^ = 0.427) followed by the 16S (Fig. 3C; *P* = 0.027, *R*^2^ = 0.207) and metagenomic (Fig. 3E; *P* = 0.036, *R*^2^ = 0.193) data. The proportion of variation explained by the first two principal components at week 2 was also the highest with the metabolite data compared to the metagenome and 16S data. At week 5, the metabolites showed significant levels of differentiation among treatments (Fig. 3B; *P* = 0.012, *R*^2^ = 0.136), while we found no significant separation based on the 16S (Fig. 3D; *P* = 0.320, *R*^2^ = 0.171) and metagenomic (Fig. 3F; *P* = 0.072, *R*^2^ = 0.153) data. For all three data sets, both the goodness of fit (*R*^2^ values) and the amount of variation explained by the first two principal components were lower at week 5 than week 2. Compositional data analysis (CoDA) via clr-transformed multiomics data sets also demonstrated a higher goodness of fit for all three data sets at week 2 than week 5, with the metabolites having a better fit at week 2 than either the metagenomics or 16S data sets. In addition, multiomics data set correlation analysis performed via the DIABLO package showed high overall correlations between all three data sets (metagenomics versus metabolomics, *r* = 0.81; metagenomics versus 16S, *r* = 0.82; metabolomics versus 16S, *r* = 0.72). The complete data sets, analyses (Jupyter notebooks), and results of all the CoDA tests are stored on GitHub (https://github.com/bryansho/PCOS_WGS_16S_metabolome/tree/master/Revision/CLR_transform).

In addition to employing operational taxonomic unit (OTU) clustering at the 97% (genus) level for 16S rRNA gene analysis, we also analyzed our data using denoised amplified sequence variants (SVs) and SVs clustered at the 97% level. The programming scripts used to generate the SVs and results for CAP analysis, random forest, and DESeq2 are included in supplementary data at https://github.com/bryansho/PCOS_WGS_16S_metabolome/tree/master/Revision/16S%20SV.

CAP analysis of the 16S data using SVs and 97% clustered SVs confirmed the OTU results: the highest level of differentiation among treatment groups was found at time 2 versus time 5 (see Table S1 at https://github.com/bryansho/PCOS_WGS_16S_metabolome/tree/master/Revision/16S%20SV). Interestingly, the goodness-of-fit was notably better for the OTU data at both time points compared to the SV or clustered SV data. A similar pattern was observed with the random forest analysis, with equal or higher preiction accuracy for the OTU data, particularly at time 2 (see Table S2 at https://github.com/bryansho/PCOS_WGS_16S_metabolome/tree/master/Revision/16S%20SV).

### Combining multiomics data sets did not improve goodness-of-fit in beta diversity analyses.

**FIG 4:**
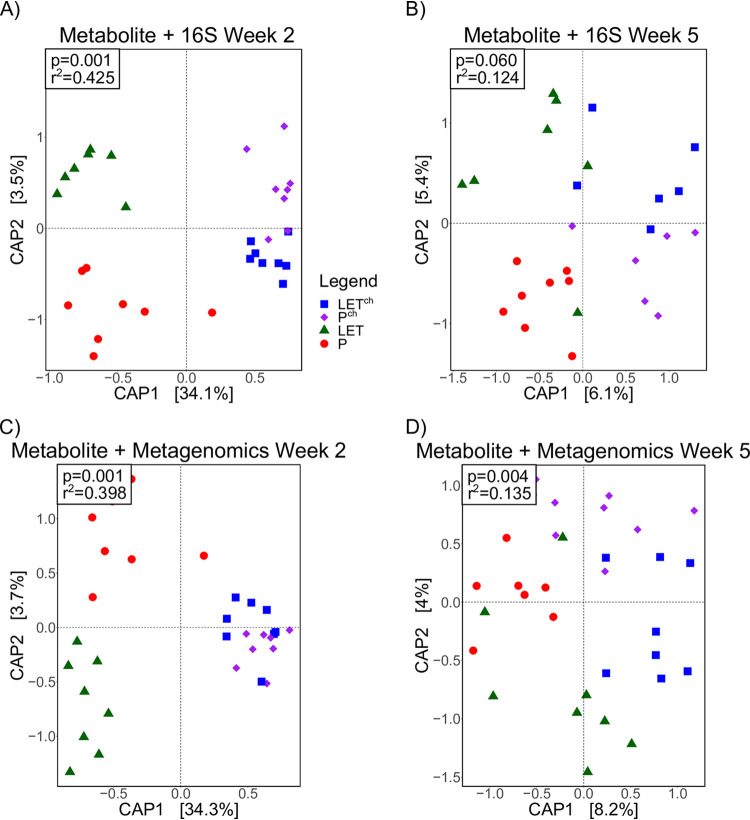
Combination of metabolomic and metagenomic data did not improve the fit of overall microbial compositional data compared to metabolomic data set alone. The cohousing study resulted in four treatment groups: placebo mice (P), letrozole mice (LET), placebo cohoused with letrozole (P^ch^), and letrozole cohoused with placebo (LET^ch^). Canonical analysis of principal (CAP) coordinates of Bray-Curtis dissimilarity among the four treatment groups, week 2 (A, C) and week 5 (B, D). Shown are the CAP1 and CAP2 coordinates, representing the two coordinates that captured the greatest amount of variation (percentage of variation is shown in brackets). (A and B) Metabolites combined with bacterial 16S rRNA gene sequences (16S); (C and D) metabolites combined with bacterial whole-genome sequencing (WGS).

CAP analyses were performed after combining the metabolomics data with the 16S and metagenomic data sets at weeks 2 and 5, respectively. At week 2, the combined metabolite and 16S data showed clear differentiation among treatment groups (Fig. 4A; *P* = 0.001, *R*^2^ = 0.425) but was not different at week 5 (Fig. 4B; *P* = 0.060, *R*^2^ = 0.124). For the combined metabolite and metagenomic data set, treatment groups were significantly different at both week 2 (Fig. 4C; *P* = 0.001, *R*^2^ = 0.398) and week 5 (Fig. 4D; *P* = 0.004, *R*^2^ = 0.135). The amount of variation explained by the first two principal components was higher at week 2 for both combined data sets. The goodness-of-fit and amount of variation explained by the principal components at week 2 were not greater than determined independently for the metabolite data set (Fig. 3A), indicating that the metabolite data set in this multiomics analysis was the main contributor to the observed treatment differentiation.

### Log_2_ fold analysis identified numerous differential metabolite abundances between treatment groups.

**FIG 5:**
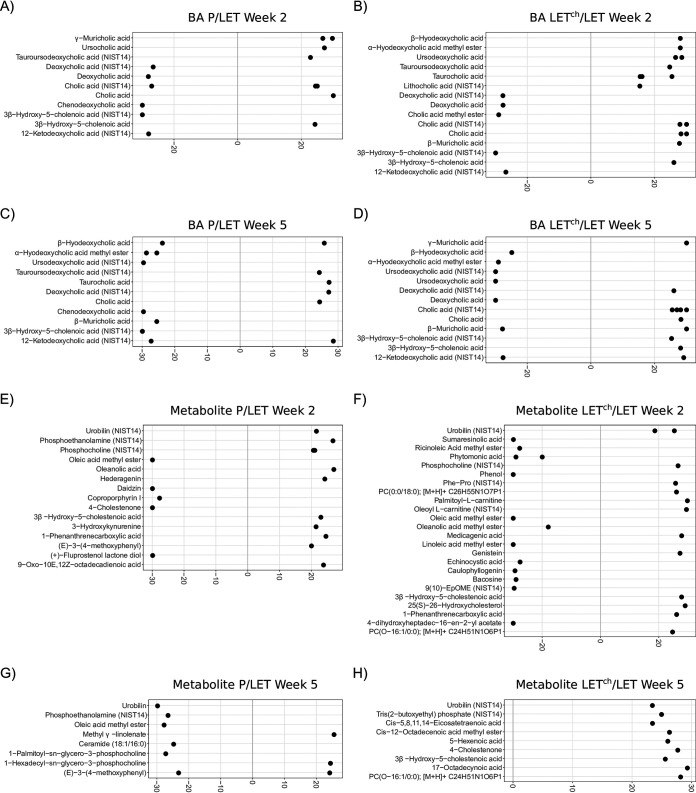
Primary and secondary BA relative abundance was altered in a pairwise comparison of P relative to LET or LET^ch^ relative to LET. The cohousing study resulted in four treatment groups: placebo mice (P), letrozole mice (LET), placebo cohoused with letrozole (P^ch^), and letrozole cohoused with placebo (LET^ch^). Results from the DESeq2 analysis were expressed as log_2_ fold change for P/LET (A, C, E, G) and LET^ch^/LET (B, D, F, H) with BAs at week 2 (A and B), BAs at week 5 (C and D), other identified metabolites at week 2 (E and F), and other identified metabolites at week 5 (G and H). The 10 BAs or metabolites with the greatest magnitude of log_2_ fold change were shown for each comparison. Positive log_2_ fold changes represent metabolites increased in P or LET^ch^ relative to LET, while negative changes represent metabolites increased in LET relative to P or LET^ch^.

To identify specific features that contributed to the difference in treatment groups, we calculated log_2_ fold ratios for the relative abundances of primary and secondary BAs and other identifiable metabolites (Fig. 5). For the BAs, the number of differential BAs was greater between LET^ch^ and LET than between P and LET, particularly at week 2 (Fig. 5A to D). We found a similar, though more pronounced, pattern with the other identified metabolites (Fig. 5E to H). Almost twice as many identifiable metabolites were differentially abundant in LET^ch^/LET compared to P/LET at week 2 (Fig. 5E and F), but this pattern was not present at week 5 (Fig. 5G and H).

### Log_2_ fold change values smaller with bacterial relative abundances compared to metabolites.

**FIG 6:**
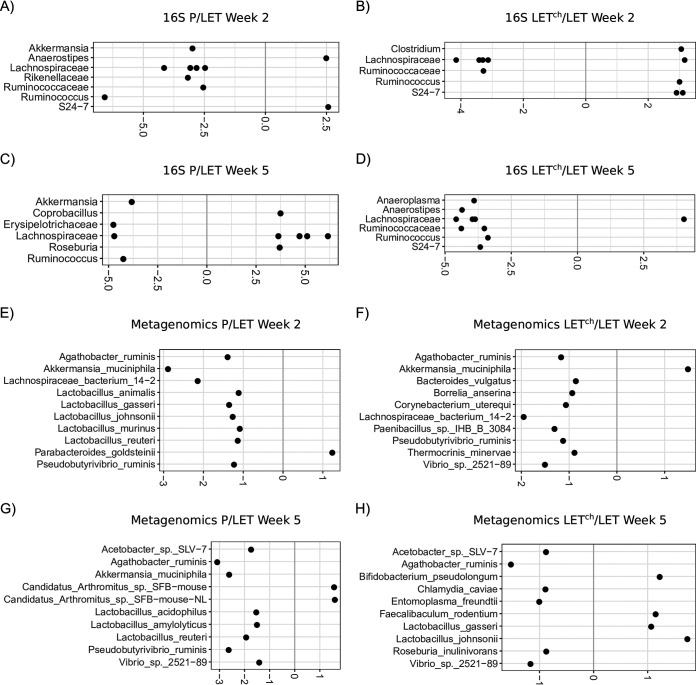
Bacterial relative abundance was altered in a pairwise comparison of P relative to LET or LET^ch^ relative to LET. The cohousing study resulted in four treatment groups: placebo mice (P), letrozole mice (LET), placebo cohoused with letrozole (P^ch^), and letrozole cohoused with placebo (LET^ch^). Results from the DESeq2 analysis were expressed as log_2_ fold change for P/LET (A, C, E, G) and LET^ch^/LET (B, D, F, H) with 16S rRNA bacterial gene sequences (16S) at week 2 (A and B), 16S at week 5 (C and D), bacterial whole-genome sequencing (WGS) at week 2 (E and F), and WGS at week 5 (G and H). The 10 bacteria (family or genus level for 16S; species level for WGS) with the greatest magnitude of log_2_ fold change were shown for each comparison. Positive log_2_ fold changes represent bacteria increased in P or LET^ch^ relative to LET, while negative changes represent bacteria increased in LET relative to P or LET^ch^.

Figure 6 shows the results of log_2_ fold analyses based on relative bacterial abundances estimated via 16S rRNA gene sequences (Fig. 6A to D) and metagenomes (Fig. 6E to H). The plots include the 10 taxa with the greatest fold change from each comparison. The fold change values ranged from −6 to +6 with the 16S OTU data, and −3 to +2 with the metagenomic data, compared with a fold change range of −30 to 30 with the metabolites (Fig. 5). In the 16S data, comparisons of week 2 and week 5 found 6 of the top 10 taxa with the highest fold change values were the same for both P/LET and LET^ch^/LET comparisons. At week 2, P/LET and LET^ch^/LET shared 6 of the same OTUs, and all but *Ruminococcus* had a similar positive or negative change in ratio. For the metagenomic data, we identified 4 bacterial species shared between week 2 and week 5 for P/LET and 2 species shared between weeks 2 and 5 in the LET^ch^/LET comparisons. Only 2 species, Akkermansia muciniphila and Pseudobutyrivibrio ruminis, were in common at week 2 between the P/LET and LET^ch^/LET comparisons. A comparison of DESeq2 results for the OTU data with those of the clustered SV 16S data found that 50% or more of the top 10 most differentially abundant OTU taxonomic groups were among the top 10 most differentially clustered SVs (see Table S3 at https://github.com/bryansho/PCOS_WGS_16S_metabolome/tree/master/Revision/16S%20SV), while most of the rest were present among the top 20 most differentially abundant taxa.

As a complement to the DESeq2 analysis, we applied a compositional data approach to detect differentially abundant taxa. The programming scripts and results of the ANCOM analyses for metabolite, 16S, and whole-genome sequencing (WGS) data sets are in supplementary data at https://github.com/bryansho/PCOS_WGS_16S_metabolome/tree/master/Revision/ANCOM.

ANCOM confirmed the results of the DESeq2 analysis for BAs and other metabolites. The majority of differentially abundant metabolite and 16S features in the DESeq2 analysis were present in the ANCOM results. For the WGS data, on the other hand, the ANCOM results returned very few differentially abundant taxa and there was minimal overlap between the WGS DESeq2 and ANCOM results.

### Multiomic data combinations improved random forest classification accuracy.

**FIG 7:**
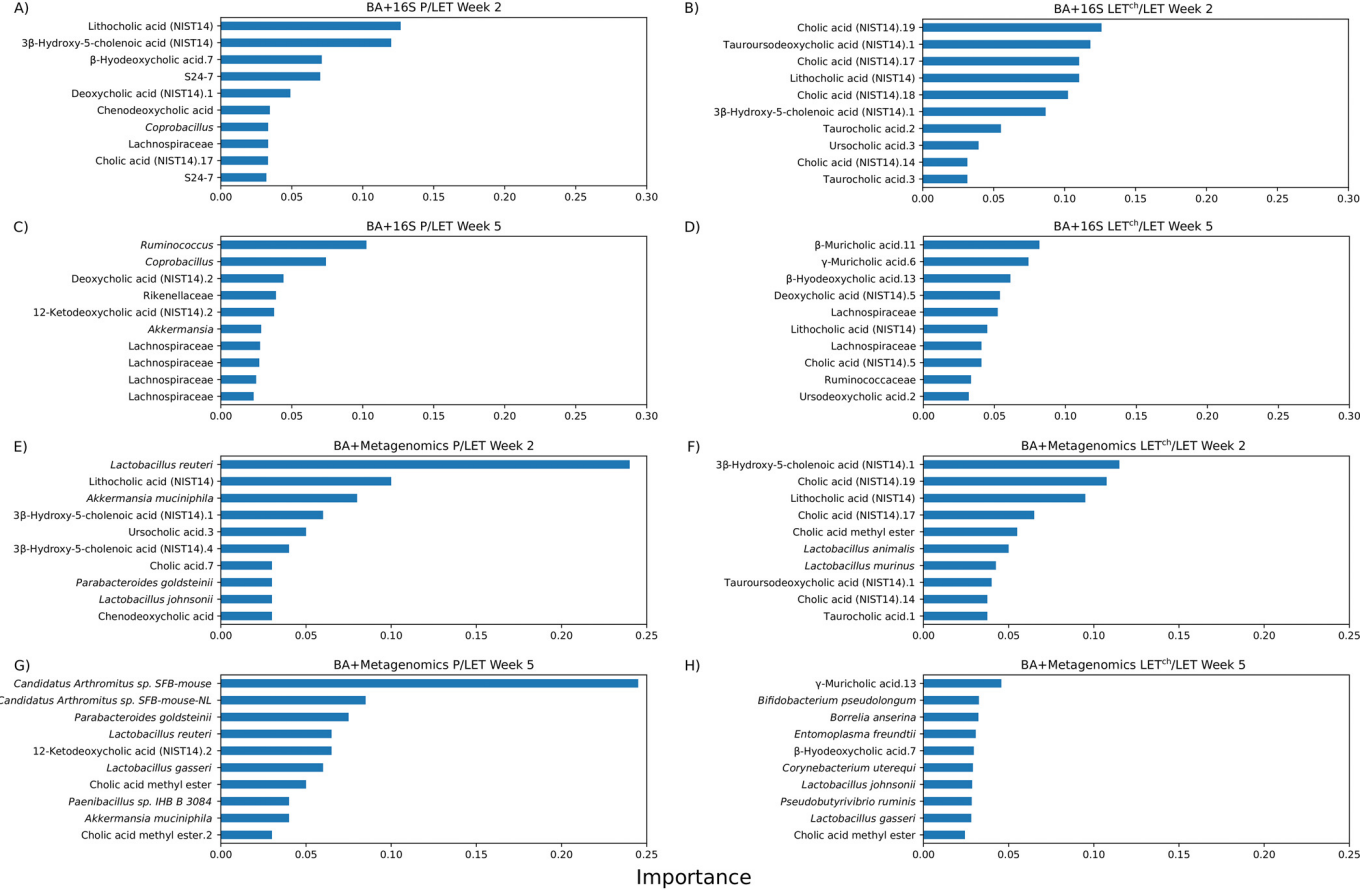
Top 10 BA and bacterial features that classify P versus LET or LET^ch^ versus LET in random forest analysis. The cohousing study resulted in four treatment groups: placebo mice (P), letrozole mice (LET), placebo cohoused with letrozole (P^ch^), and letrozole cohoused with placebo (LET^ch^). The graphs show Gini importance scores which indicate the relative importance of a particular feature (BA or bacteria) in the classification result. Results are shown for P/LET (A, C, E, G) and LET^ch^/LET (B, D, F, H) with BA + bacterial 16S rRNA gene sequencing (16S) at week 2 (A and B), BA + 16S at week 5 (C and D), BA + whole-genome sequencing (WGS) at week 2 (E and F), and BA + WGS at week 5 (G and H).

Table 1 shows the results of random forest analysis classification of P versus LET and LET^ch^ versus LET comparisons at weeks 2 and 5 for independent and combined data sets. In general, the week 2 data sets were better able to classify treatments than the week 5 data sets, and classification accuracy was highest with LET^ch^ versus LET. Among independent data sets, the 16S data set had the highest classification accuracy, while the combination of BA and metagenomic data resulted in the best overall accuracy for both P-versus-LET and LET^ch^-versus-LET comparisons. An analysis of features that contributed the most to the accuracy of the combined multiomics random forest analyses is shown in Fig. 7. Specifically, the 10 features with the highest Gini importance scores are shown; their removal from the data set had the greatest effect on the ability to classify between the treatment conditions. In the week 2 samples, BAs were the majority of the top 10 features with the highest Gini importance. In the LET^ch^-versus-LET comparisons, all of the most important classification features were BAs when they were combined with 16S data (Fig. 7B), while 8 of the 10 most important features were BAs when they were combined with metagenomic data (Fig. 7F). At week 2, 6 out of the 10 most important features were BAs in the P-versus-LET comparisons at week 2 for both the BA + 16S combination (Fig. 7A) and the BA + metagenome combination (Fig. 7E). In the week 5 samples, the case was reversed: with one exception (Fig. 7D, BA +16S LET^ch^ versus LET), the bacterial taxa dominated the top 10 importance features.

**TABLE 1:** Results of random forest analyses with single and combined multiomics data sets^*e*^

Comparisons	Accuracy^*a*^	OOB score^*b*^	Mean accuracy^*c*^	AUC^*d*^
Wk 2				
P vs LET (BA)	16.67%	50.00%	40.00%	0.00%
P vs LET (16S)	83.33%	20.00%	70.00%	66.66%
P vs LET (WGS)	66.67%	80.00%	70.00%	100.00%
LET vs LET^ch^ (BA)	66.67%	80.00%	80.00%	100.00%
LET vs LET^ch^ (16S)	100.00%	93.75%	90.00%	100.00%
LET vs LET^ch^ (WGS)	66.67%	40.00%	40.00%	66.67%
P vs LET (BA + 16S)	50.00%	50.00%	60.00%	66.66%
P vs LET (BA + WGS)	100%	60.00%	60.00%	100.00%
LET vs LET^ch^ (BA + 16S)	100.00%	100.00%	90.00%	100.00%
LET vs LET^ch^ (BA + WGS)	100%	90.00%	80.00%	100.00%

Wk 5				
P vs LET (BA)	50.00%	80.00%	80.00%	66.67%
P vs LET (16S)	50.00%	40.00%	60.00%	55.55%
P vs LET (WGS)	66.67%	90.00%	70.00%	55.56%
LET vs LET^ch^ (BA)	66.67%	80.00%	70.00%	44.45%
LET vs LET^ch^ (16S)	40.00%	30.00%	73.33%	16.66%
LET vs LET^ch^ (WGS)	66.67%	60.00%	70.00%	88.89%
P vs LET (BA + 16S)	50.00%	30.00%	60.00%	66.66%
P vs LET (BA + WGS)	83.30%	60.00%	90.00%	78.00%
LET vs LET^ch^ (BA + 16S)	40.00%	77.77%	73.33%	83.33%
LET vs LET^ch^ (BA + WGS)	83.30%	30.00%	50.00%	88.90%

a RF accuracy = percentage of correctly classified samples.

b Out-of-bag (OOB) score = percentage of correctly classified samples based on unselected samples from the bootstrap sampling method.

c Mean accuracy = average accuracy of a 5-fold cross-validation.

d Area under the curve = measurement of the performance of a binary classifier.

e Abbreviations: P, placebo; LET, letrozole; LET^ch^, LET cohoused with placebo; BA, bile acids; 16S, 16S rRNA gene sequences; WGS, whole-genome sequencing data.

### Analyses revealed time- and treatment-specific patterns of correlations between bacteria and BAs.

**FIG 8:**
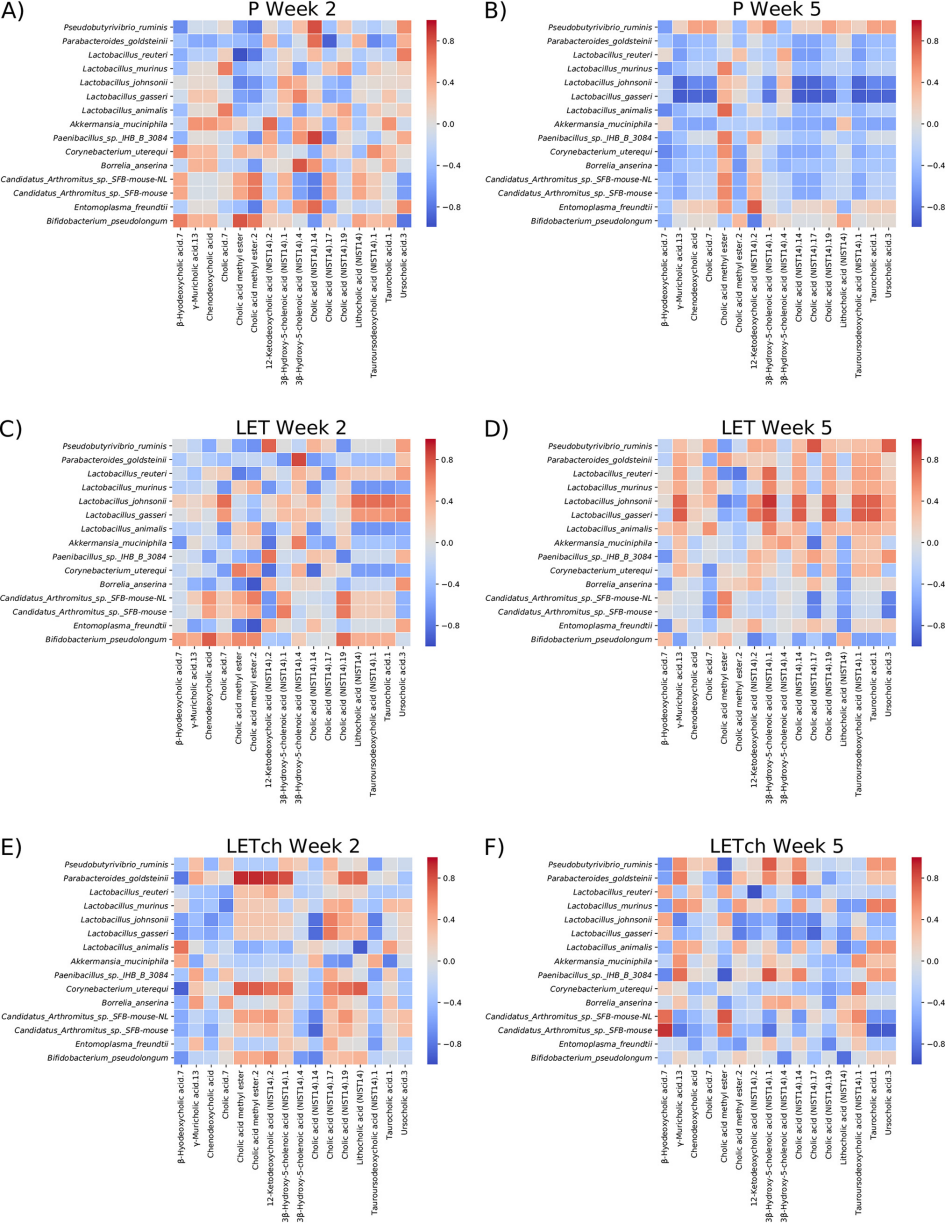
Heatmaps of Spearman-rank correlation values between clr-transformed BA and bacterial abundances. Red indicates positive correlation values, while blue indicates negative correlation values. The BA and bacterial features chosen for analysis were selected based on DESeq2 analyses. The cohousing study resulted in four treatment groups: placebo mice (P), letrozole mice (LET), placebo cohoused with letrozole (P^ch^), and letrozole cohoused with placebo (LET^ch^). (A and B) P, (C and D) LET, and (E and F) LET^ch^ at week 2 or 5, respectively.

The heatmaps in Fig. 8 illustrate the results of correlations between BAs and bacterial species identified in the metagenomic data. For all the treatment groups (P, LET, and LET^ch^), we observed more than twice the number of strong correlations (*P* ≥ |0.8|; dark red or blue squares) in samples collected at week 2 than in those collected at week 5. Moreover, none of the strongest correlations detected between BAs and bacterial species at week 2 were detectable at week 5. For example, in the week 2 P samples, one cholic acid metabolite was positively correlated with 6 different bacterial species. However, this same metabolite was weakly or even negatively correlated with the same 6 species at week 5. Similar patterns could be observed in the LET (e.g., γ-muricholic acid) and LET^ch^ (e.g., cholic acid.7) treatment groups. The patterns of correlations between bacteria and BAs also differed considerably among treatment groups. Of the 10 strongest correlations between BAs and bacterial species in the P samples at week 2, 8 were not detectable in the LET samples. Similarly, none of the 10 strongest correlations in LET^ch^ at week 2 were correlated in LET at the same time point.

## DISCUSSION

Multiomics methods improved the fit of gut microbiome samples to the experimental treatment conditions, increased the resolution of the time-dependent changes in the gut microbiome, and identified potential targets for future mechanistic studies. The untargeted metabolomic analysis proved especially useful for distinguishing treatment groups. At time zero of the longitudinal sampling, there were no detectable differences among treatment groups (Fig. 2A), but by week 1 the untargeted metabolomic data indicated clear patterns of differentiation (Fig. 2B). By week 2, all three data sets showed a pattern of differentiation, but the metabolite data were the best fit to the treatment conditions (Fig. 2C). Analysis of the untargeted metabolites from the cohousing samples also revealed a pattern of early divergence of both P^ch^ and LET^ch^ from the P and LET microbiome samples, which were also distinct from one another. Furthermore, effects of cohousing on the LET^ch^ gut microbiome did not result in a “placebo-like” state; rather, cohousing resulted in a third state, distinct from both P and LET conditions, indicating that the gut microbiome does not have to “return” to a healthy state to have protective effects.

In addition to OTU clustering for the 16S data, we also performed analyses using denoised SVs and clustered SVs (see supplementary data at https://github.com/bryansho/PCOS_WGS_16S_metabolome/tree/master/Revision/16S%20SV). The beta diversity patterns with SVs and clustered SVs were very similar to the OTU results, though the goodness-of-fit in the CAP analysis was better with the OTUs than either the SVs or clustered SVs, which was somewhat surprising given that the OTUs and clustered SVs were both clustered at the 97% (genus) level. We also note that the accuracy of the random forest analyses with the OTU data was more robust than that with the SV data in terms of accuracy. While not identical, the DESeq2 analysis with the clustered SVs had considerable overlap with the OTU analysis in terms of the differentiated taxa. Because the log_2_ fold analysis implemented by DESeq2 may be prone to false positives, we also reanalyzed feature-level differentiation with the metabolite, 16S, and WGS data with ANCOM, a compositional data analysis approach. The DESeq2 and ANCOM results for the metabolite data were very similar, particularly for the BAs. We also observed a high degree of similarity with the 16S data between the DESeq2 and ANCOM results. The 16S results from the ANCOM approach found many additional differentiated taxa compared with DESeq2, including many uncultured taxa, but the same identified bacterial taxa differentiated in DESeq2 results were also present in the ANCOM results. The WGS results, on the other hand, were quite dissimilar between the DESeq2 and ANCOM results, though it is important to note that there was a very low level of feature differentiation in both the DESeq2 and ANCOM results.

The time-dependent nature of the shift in the gut microbiome due to both LET treatment and cohousing was apparent in all the ‘omics data sets. Untargeted metabolomics, 16S, and shotgun metagenomics showed stronger separation at week 2 samples than week 5 samples, with the metabolite data being the best fit to treatment at both time points (Fig. 3). These results further confirm the importance of longitudinal sampling. Had we waited until the end of the experiment to collect fecal samples, or sampled at only a single time point, our interpretation of the results would have been affected. Overall, three clear patterns emerged from the longitudinal multiomics analyses: (i) a shift in overall gut metabolite diversity was detectable prior to the shift in bacterial diversity (16S or metagenomics), (ii) a detectable shift in gut metabolites occurred by the first week of the study, and (iii) both P^ch^ and LET^ch^ were distinct from the P and LET samples, respectively, and were also more similar to one another than either was to the P and LET samples. The early community-wide shift in metabolite diversity in the LET samples suggests that these changes began even earlier, perhaps immediately after letrozole treatment and the resultant increase in testosterone levels. Further, our results suggest that metabolites transferred quickly between P^ch^ and LET^ch^ mice, likely via coprophagy. Given these patterns, we hypothesize that host-related changes led to changes in gut microbial diversity, though future experimentation will be needed to understand the timing and nature of these changes.

While little is currently known about the role specific BAs have in modulating host-microbe interactions, it is well known that BAs can affect the growth of gut bacteria and that they are chemically modified by gut bacteria. BAs have been reported to promote the growth of BA-metabolizing bacteria and have strong antibiotic properties (34) and favor resistant bacteria such as *Lactobacillus* and *Bifidobacterium* (35). Numerous gut bacteria including members of *Lactobacillus*, *Bifidobacterium*, and *Clostridium* also express bile-salt hydrolase (BSH) enzymes, enabling them to deconjugate BAs (36). In addition, *Clostridium* species can metabolize secondary BAs (36). Interestingly, our log_2_ fold analysis found that many species within these bacterial genera (e.g., *Lactobacillus*, *Bifidobacterium*, *Akkermansia*, and *Clostridium*) were differentially abundant between P and LET as well as LET and LET^ch^, particularly at week 2 (Fig. 6). A recent study by Tian et al. measured the effects of BAs on the growth and survival of four common gut bacteria and found that different BAs had bacterium-specific effects (37). Interestingly, the BAs they tested in the study, lithocholic acid (LCA), deoxycholic acid (DCA), taurocholic acid (TCA), and TUDCA, were identified as differentially abundant in log_2_ fold comparisons between P/LET and LET^ch^/LET (Fig. 5) and had high Gini importance scores in the random forest analyses, particular at week 2 (Fig. 7A and B). In addition, our study identified strong correlations between 3 of these BAs (LCA, TCA, and TUDCA) and various gut bacteria in our study (Fig. 8). Furthermore, the magnitude of these correlations varied considerably among treatment groups. For example, in the LET samples at week 2 LCA, TCA, and TUDCA were highly positively correlated with Lactobacillus johnsonii and Lactobacillus gasseri and strongly negatively correlated with Lactobacillus murinus and Lactobacillus animalis (Fig. 8C). At the same time point, there were no correlations detected between these three BAs and these *Lactobacillus* species in P samples (Fig. 8A) and only strong negative correlations between TUDCA and L. johnsonii and L. gasseri in LET^ch^ (Fig. 8E). Interestingly, a recent study of the effects of BAs on the neonatal microbiome found that administration of specific BAs to mice via oral gavage had a particularly strong effect on members of *Lactobacillus* and that this effect was highly species dependent (38). L. johnsonii, which carries BSH enzymes of different specificities (39), was strongly affected by the administration of multiple different BAs, while the BSH-negative *L. murinus* was unaffected. Two other striking patterns were the strong positive correlations in the week 2 LET^ch^ samples between the same four BAs and Parabacteroides goldsteinii and, to a lesser extent, Corynebacterium uterequi (Fig. 8E), correlations not observed in either LET or P samples. Species of *Parabacteroides* have been suggested as potential therapeutics for obesity-related disorders and have been shown to metabolize BAs (40).

Strengths of this study include the use of multiple ‘omics approaches for assessing changes in the gut microbiome correlated with LET and cohousing, longitudinal sampling, and a diet-independent mouse model that recapitulates many phenotypes of PCOS. Limitations of the study include how representative the experimentally induced LET mouse model is of PCOS in women and the relatively small sample size. Despite these caveats, our study provided additional evidence that changes in primary and secondary BAs may be associated with PCOS. One study recently demonstrated that levels of secondary BAs such as glycodeoxycholic acid and TUDCA were lower in women with PCOS than in women without PCOS (20). Interestingly, we also observed a lower ratio of TUDCA in LET versus P mice as well as a higher ratio of TUDCA in LET^ch^ versus LET mice (Fig. 5A to C). Given that supplementation with these BAs was shown to be protective against developing a PCOS-like phenotype in mice (20), further studies will be needed to determine whether treatment with specific BAs is protective in the letrozole-induced PCOS-like mouse model, what mechanisms are involved in this protective effect, and whether these findings can be translated to develop novel therapies for women with PCOS. Somewhat surprisingly given the importance of BAs in host physiology, much remains to be discovered concerning the mediation of BA signaling by host-microbe interactions. With regard to PCOS, this will necessitate mechanistic studies in PCOS-like animal models focused on the role of sex steroids in regulating BA production in the liver, BA metabolism by gut microbes, and BA signaling in the enterohepatic system.

## MATERIALS AND METHODS

### Letrozole-induced PCOS-like mouse model.

Details on the mouse model and cohousing experimental design were described previously (26). Briefly, C57BL/6NHsd female mice (Envigo) were housed in a vivarium under specific-pathogen-free conditions with *ad libitum* access to water and food. Placebo or 3-mg letrozole pellets (50 μg/day; Innovative Research of America) were implanted subcutaneously into 4-week-old mice for 5 weeks. Throughout the experiment, mice were housed two per cage in three separate housing arrangements. This resulted in four treatment groups: placebo mice (P), letrozole mice (LET), placebo cohoused with letrozole (P^ch^), and letrozole cohoused with placebo (LET^ch^) (*n* = 8 mice/group). All animal procedures in the experiment were approved by the University of California, San Diego Institutional Animal Care and Use Committee (protocol S14011).

### Fecal sample collection, DNA isolation, and 16S rRNA gene sequencing.

Fecal sample collection, DNA extraction, PCR, and 16S rRNA library sequencing were performed as previously described (26). Fecal samples were collected prior to pellet implantation and once per week for the duration of the experiment. Fecal samples were frozen immediately after collection and stored at −80°C. DNA was extracted from the samples using the DNeasy PowerSoil kit (Qiagen) according to the manufacturer’s protocol and stored at −80°C. PCR amplification was performed for the V4 hypervariable region of the 16S rRNA gene with primers 515F and 806R (41). The reverse primers contained unique 12-bp Golay barcodes that were incorporated into the PCR amplicons (41). Amplicon sequence libraries were prepared at the Scripps Research Institute Next Generation Sequencing Core Facility and sequenced on an Illumina MiSeq.

### Bioinformatics and statistical analysis of 16S rRNA gene sequences.

Processing of sequences and OTU picking were performed using accessory scripts from QIIME version 1.9.1 (42). Only forward reads were used from the Illumina sequencing data. Barcodes were extracted from the Illumina 16Ss fastq file using extract_barcodes.py, and the data were demultiplexed and quality filtered using split_libraries_fastq.py with default parameters. OTU picking was performed using a *de novo* approach with the pick_de_novo_otus.py script, using Greengenes 13.8 as the reference database (43). The OTU table was then parsed using filter_otus_from_otu_table.py, and any OTUs not present in at least 25% of samples were removed prior to downstream analysis.

### Fecal metabolite extraction and LC-MS/MS.

Individual fecal samples were weighed to ensure they weighed at least 0.01 g per fecal sample. The fecal samples were transferred to 2-ml vial inserts to which a 1:10 volume of methanol (Optima LC/MS-grade methanol, 67-56-1; Fisher Scientific) diluted in water 70:30 (Optima LC/MS-grade water, 7732-18-5; Fisher Scientific) was added. The sample was then homogenized in a Qiagen TissueLyser and allowed to extract overnight at room temperature. After extraction, samples were briefly vortexed and incubated for 1 h at room temperature before centrifugation at 10,000 × *g* for 30 s. Liquid chromatography (LC) was performed with Thermo Scientific UltraMate 3000 Dionex. High-performance liquid chromatography (HPLC) was performed using a Phenomenex (Torrance, CA, USA) Luna 5-μm C_18_(2) HPLC column (2.0 mm by 250 mm), and ultrahigh-performance liquid chromatography (UPLC) was performed using a Phenomenex Kinetex 2.6-μm C_18_ (30 by 2.10 mm) column with 20 μl of the extractions from the fecal pellets. A linear water–acetonitrile gradient (from 98:2 to 2:98 water-acetonitrile) containing 0.1% formic acid was used (HPLC, 54-min gradient; UPLC, 14-min gradient) with a flow rate of 0.2 ml min^−1^ for the HPLC analysis and 0.5 ml min^−1^ for the UPLC analysis. Tandem mass spectrometry (MS/MS) was performed using a Bruker Daltonics Maxis quadrupole time of flight (qTOF) mass spectrometer equipped with a standard electrospray ionization source. Tuning of the mass spectrometer was done by infusion of Tuning Mix ES-TOF (Agilent Technologies) at a 3 μl min^−1^ flow rate. Lock mass internal calibration used a wick saturated with hexakis (1H,1H,3H-tetrafluoropropoxy) phosphazene ions (Synquest Laboratories, *m/z* 922.0098) located within the source for accuracy. The mass spectrometer was operated in data-dependent positive-ion mode, automatically switching between full-scan MS and MS/MS acquisitions for both the HPLC and UPLC analyses. Full-scan MS spectra (*m/z* 50 to 2,000) were acquired in the TOF, and the top 10 most intense ions in a particular scan were fragmented using collision-induced dissociation at 35 eV for +1 ion and 25 eV for +2 ions in the collision cell.

### Bioinformatics analysis of metabolites.

Molecular networks were created by using the GNPS database online workflow at http://gnps.ucsd.edu, and the data set was used to search various MS/MS libraries available in the GNPS database by using the same workflow. The data set is available to the public at the online MassIVE repository of the GNPS database under MassIVE ID number MSV000081524. The molecular network used for analysis is available at https://gnps.ucsd.edu/ProteoSAFe/status.jsp?task=d74cab73cc344821a59979f58269f3a8.

Features were quantified using the mzMine-based feature finding algorithm with qTOF presets on the GNPS workflow. The features in the table were then filtered by removing features that were present in fewer than four samples, which removed approximately 50% of the total features. Metabolite annotations are based on MS/MS matches in the GNPS libraries and are therefore considered level two according to the metabolomics standards initiative (44).

### Shotgun metagenomic sequencing and preprocessing.

Eight hundred nanograms of genomic DNA isolated from week 2 and week 5 samples was sonicated using an E220 focused ultrasonicator (Covaris) to produce 400-bp fragments which were purified using Agencourt AMPure XP beads (Beckman Coulter). A KAPA Hyper Prep kit (Kapa Biosystems) was used to prepare Illumina libraries following the manufacturer's instructions. Libraries were quality checked for their size and concentration with electrophoresis using a high-sensitivity D1000 kit on a 2200 TapeStation (Agilent). Prepared samples were sequenced by the Center for Advanced Technology at the University of California, San Francisco, using an Illumina NovaSeq sequencer set to 150-bp paired-end reads. This produced an average of 109,084,767 reads per sample. Sequences from metagenomes were trimmed and filtered based on quality score, read length, and number of ambiguous nucleotides (N) using Fastp (version 0.19.6) (45). Adapters and poly(G) tails (NovaSeq’s no-signal indication) were automatically detected and removed. This preprocessing step removed <1% of reads per sample, resulting in an average read length of 146 bp and a Q20 of 99.1%. Preprocessed paired-end reads were then mapped using Bowtie2 (version 2.2.6) to the mouse host genome (University of California, Santa Cruz Mus musculus genome; mm10) to remove host contamination (46). Mapped reads were removed with SAMtools (version 1.5), and unmapped reads were reconstructed to paired-end reads with BEDTools (version 2.25.0) (46, 47).

### Bioinformatics analysis of metagenome data.

Taxonomic identification was performed using Centrifuge (version 1.0.3) against the NCBI nonredundant sequence database (48), and archaea, eukaryotes, and viral results were removed. Bacterial features (18,464) were passed through a filter that required species to be present in 90% of all samples and have an abundance of greater than 0.00001%, resulting in 3,539 bacterial species for analysis.

### Statistical analysis of metabolites, 16S rRNA genes, and metagenomes.

Analysis of the similarity among treatment groups was done via CAP using Bray-Curtis dissimilarity through the R package (version 1.26.1) (49). Differences among treatment groups were determined by permutational multivariate analysis of variance (PERMANOVA) using the Python package Scikit-bio (version 0.5.5). Differential expressions of features were determined through the R package DESeq2 (version 1.18.1) using Wald’s test to find the log_2_ fold expression levels between treatment groups (50). Comparisons were made between P and LET treatments and LET and LET^ch^ treatments for each time point (week 2 and week 5). To test for the effects of potential statistical artifacts in the analysis of relative abundance data due to its inherent compositional nature, we transformed all the ‘omics data sets independently using the centered log-ratio (clr). Specifically, we clr transformed the processed and filtered metagenomic, 16S, and metabolite data sets at time points 2 and 5 using Python package Scikit-bio (version 0.5.5) with multiplicative replacement to adjust for zero values. Euclidean distances calculated using the clr-transformed data were then subjected to NMDS (nonmetric multidimensional scaling) ordination and PERMANOVA tests in R package vegan (version 2.5-6). In addition, correlations between the metagenomic, metabolite, and 16S data sets were assessed with the DIABLO (Data Integration Analysis for Biomarker discovery using a Latent cOmponents) framework in R package mixOmic (version 6.10.9). Treatment conditions and time points were used as conditions in DIABLO for evaluation.

### Multiomics analyses.

To detect potential associations between the members of the gut microbial community and specific metabolites, two multiomics feature tables were created: (i) metabolomes combined with 16S rRNA sequencing and (ii) metabolomes and metagenomes. Multiomics data were analyzed via CAP based on Bray-Curtis dissimilarities through Phyloseq (version 1.26.1) (49). BAs and bacterial species with the largest log_2_ fold magnitude as indicated by DESeq2 were used as features in a random forest supervised learning model analysis via Scikit-learn (version 0.20.1) in Python (50, 51). All models were optimized to have the lowest out-of-bag error. Following the same comparison structures in the DESeq2 analysis, the top 10 features with the highest Gini importance index for all comparisons were selected for a correlation analysis. To combine multiomics data sets at very different scales for correlation analysis, we transformed the data sets independently using the centered log-ratio (clr) approach. Zero-replacement was performed with the pseudocount method from the R package zCompositions version 1.3.3. Spearman-rank correlation analysis was performed in Python pandas (version 0.25.1) with clr-transformed BA counts and clr-transformed bacterial species counts from the metagenomic data for P, LET, and LET^ch^ at weeks 2 and 5. Heatmaps were generated using the Python seaborn package (version 0.9.0) (52, 53).

### Data availability.

16S rRNA gene sequences used in this study are available via the European Nucleotide Archive (study accession number PRJEB29583). Shotgun metagenomes are available via the European Nucleotide Archive (study accession number PRJEB40312). Metabolomics data are available online in the MassIVE repository of the GNPS database under MassIVE ID number MSV000081524. The metadata and all code used to analyze and visualize data are available at https://github.com/bryansho/PCOS_WGS_16S_metabolome.
